# Measuring the impact of migraine: a systematic review of the measurement properties of MIDAS, HIT-6, and MSQoL

**DOI:** 10.1055/s-0046-1829149

**Published:** 2026-10-27

**Authors:** Shilpa Prasad, Rathnasabapathi Elaiyappan, Venkatesan Hariram, Sitharthan Ramalingam, Vettrivel Arul

**Affiliations:** 1 Vinayaka Missions Research Foundation (VMRF-DU), Vinayaka Mission's Homoeopathic Medical College and Hospital (VMHMCH), Department of Case Taking and RepertorySalem TNIndia.; 2 Vinayaka Missions Research Foundation (VMRF-DU), Vinayaka Mission's Homoeopathic Medical College and Hospital (VMHMCH), Department of PediatricsSalem TNIndia.; 3 Vinayaka Missions Research Foundation (VMRF-DU), Vinayaka Mission's Homoeopathic Medical College and Hospital (VMHMCH), Department of Practice of MedicineSalem TNIndia.; 4 National Homoeopathy Research Institute in Mental Health, Department of Practice of MedicineKottayam KLIndia.; 5 Vinayaka Missions Research Foundation (VMRF-DU), Vinayaka Mission's Homoeopathic Medical College and Hospital (VMHMCH), Department of Community MedicineSalem TNIndia.

**Keywords:** Migraine Disorders, Disability Evaluation, Surveys and Questionnaires, Quality of Life, Psychometrics

## Abstract

**Background:**

Migraine is a leading cause of global disability. Valid, reliable instruments are essential for quantifying its impact. The Migraine Disability Assessment (MIDAS), Headache Impact Test (HIT-6), and Migraine-Specific Quality-of-Life questionnaire (MSQoL) are widely used, yet their comparative measurement properties across populations remain insufficiently characterized.

**Objective:**

To systematically review and compare the measurement properties of MIDAS, HIT-6, and MSQoL following the Consensus-Based Standards for the Selection of Health Measurement Instruments (COSMIN) methodology.

**Methods:**

This systematic review followed the COSMIN guidelines for systematic reviews of outcome measurement instruments and was reported per the Preferred Reporting Items for Systematic Reviews and Meta-Analysis (PRISMA)-COSMIN guideline. In total, seven databases were searched from study inception to January 31, 2025, restricted to English-language full-text studies. Methodological quality was appraised with the COSMIN Risk of Bias checklist; results were rated against criteria for good measurement properties, and evidence was graded using a modified Grading of Recommendations, Assessments, Development, and Evaluation (GRADE) approach.

**Results:**

Of 2,674 records, 22 studies (20,455 participants) were included. All three instruments showed sufficient internal consistency and construct validity. The HIT-6 showed sufficient ratings across all properties (moderate-to-high quality evidence). Also, the MIDAS showed sufficient core properties but limited responsiveness and inconsistent reliability across cultural versions. Finally, MSQoL showed sufficient structural validity and internal consistency but insufficient test-retest reliability.

**Conclusion:**

Both HIT-6 and MIDAS can be recommended for measuring migraine impact (Category A), whereas MSQoL shows potential but requires further reliability and responsiveness research (Category B). A combined approach is recommended for comprehensive migraine burden evaluation.

## INTRODUCTION

Headache disorders are the most prevalent neurological conditions worldwide, affecting nearly 40% of the global population—an estimated 3.1 billion people in 2021—and rank among the leading causes of years lived with disability across all age groups. 1 Tension-type headache is the most prevalent headache disorder, followed closely by migraine, which carries the greatest disability burden. Chronic headache disorders, including chronic migraine (≥ 15 headache days/month), form a smaller but disproportionately disabling subgroup.

The burden of migraine is pronounced in regional settings; in India prevalence approximates 25%, exceeding the global estimate of 14.7%. 2 Migraine accounts for an estimated 45.1 million disability-adjusted life years annually, 3 with substantial economic consequences. 4 Despite this, more than half of affected individuals never consult a healthcare professional, 5 and over ⅔ receive no treatment, 6 perpetuating underrecognition across diverse health systems.

Although often used interchangeably, the three instruments examined here measure related but distinct constructs. First, the Migraine Disability Assessment (MIDAS) quantifies migraine-related disability—productive days lost across paid work, household work, and social activities over 90 days. Second, the Headache Impact Test (HIT-6) measures the broader impact of headache over 4 weeks regarding pain, social, role, cognitive, psychological, and vitality domains, and has been validated across primary and secondary headache disorders. Finally, the Migraine-Specific Quality-of-Life questionnaire (MSQoL) captures migraine-specific health-related quality-of-life (QoL) across role-restrictive, role-preventive, and emotional-function domains. Whereas HIT-6 was designed for headache broadly, MIDAS and MSQoL were developed specifically for migraine, making the instruments complementary rather than interchangeable.

Given cultural, linguistic, and demographic variation globally, a critical and systematic examination of the measurement properties of these instruments is essential to guide optimal tool selection. This review systematically evaluates and compares the measurement properties of MIDAS, HIT-6, and MSQoL using the Consensus-Based Standards for the Selection of Health Measurement Instruments (COSMIN) methodology, providing evidence to inform clinical practice and future research.

## METHODS

### Study design and reporting

This systematic review of measurement properties was conducted following the COSMIN guideline for systematic reviews of outcome measurement instruments 7 8 and reported per the Preferred Reporting Items for Systematic Reviews and Meta-Analysis (PRISMA)-COSMIN for Outcome Measurement Instruments guideline. 9 The 2020 PRISMA flow diagram documented study selection. Prospective registration on the International Prospective Register of Systematic Reviews (PROSPERO) was not completed, as the synthesis was finalized prior to submission; this is transparently disclosed per their terms of use.

### Research question

The research question followed the Population, Intervention, Comparison, and Outcome (PICO) framework, in which population (P) were adults (≥ 18-years-old) with migraine or other primary headache disorders; the intervention included index instruments (I) such as MIDAS, HIT-6, and MSQoL; the comparison (C) were reference standards (headache-diary measures, physician assessment) or head-to-head comparison between the index instruments; and the outcomes (O) were COSMIN measurement properties: content validity, structural validity, internal consistency, cross-cultural validity, reliability, measurement error, criterion validity, hypotheses testing for construct validity, and responsiveness.

### Search strategy

A comprehensive search was conducted across seven databases from inception to 31 January 2025: PubMed, Cochrane Library, Embase, CINAHL, ScienceDirect, SpringerLink, and Wiley Online Library. Reference lists and relevant regional journals were hand-searched to maximise sensitivity. Searches combined Medical Subject Headings (MeSH) and free-text terms: ( *Migraine Disorders* [MeSH] OR *Migraine* OR *Chronic Headache* OR *Tension-Type Headache* ) AND ( *MIDAS* OR *HIT-6* OR *MSQoL* OR *Migraine Specific Quality of Life* ) AND ( *Psychometrics* [MeSH] OR *Reliability and Validity* [MeSH] OR *Internal Consistency* OR *Cronbach* OR Measurement Properties OR *Responsiveness* ).

### Eligibility criteria

#### *Inclusion*

- The inclusion criteria were adults with migraine or other primary headache disorders; English-language full-text publications; studies evaluating one or more measurement properties of MIDAS, HIT-6, or MSQoL.

#### *Exclusion*

- The exclusion criteria were unrelated outcomes; insufficient methodological data; abstracts, dissertations, and non-peer-reviewed materials. Finally, the English-language restriction is acknowledged as a limitation.

### Risk of bias assessment

Two investigators independently appraised each study using the COSMIN Risk of Bias checklist. 10 Each measurement property was rated very good, adequate, doubtful, or inadequate, with an overall per-property rating derived using the “worst-score-counts” principle. Content validity was appraised following the COSMIN content-validity methodology. 11

### Evidence synthesis and grading

Results were rated against criteria for good measurement properties as sufficient (+), insufficient (−), or indeterminate (?). The noted thresholds were Cronbach's α ≥ 0.70 (internal consistency); intraclass correlation coefficient (ICC) or weighted Kappa ≥ 0.70 (reliability); confirmatory factor analysis with adequate fit (structural validity); ≥ 75% of results consistent with hypotheses (construct validity); correlation or area under the curve (AUC) ≥ 0.70 (criterion validity, responsiveness).

Furthermore, results were pooled per property per instrument, evidence was graded (high/moderate/low/very low) using a modified Grading of Recommendations Assessments, Development, and Evaluation (GRADE) approach, and each instrument received an overall recommendation: Category A (recommended), B (potential, further research needed), or C (not recommended). 8

## RESULTS

### Study selection

A total of 2,674 records were identified. After duplicate removal and eligibility screening, 22 studies were included (21 from database searching plus one Brazilian MIDAS validation added through citation searching during revision). The selection process is shown in Figure 1 .

**Figure 1:**
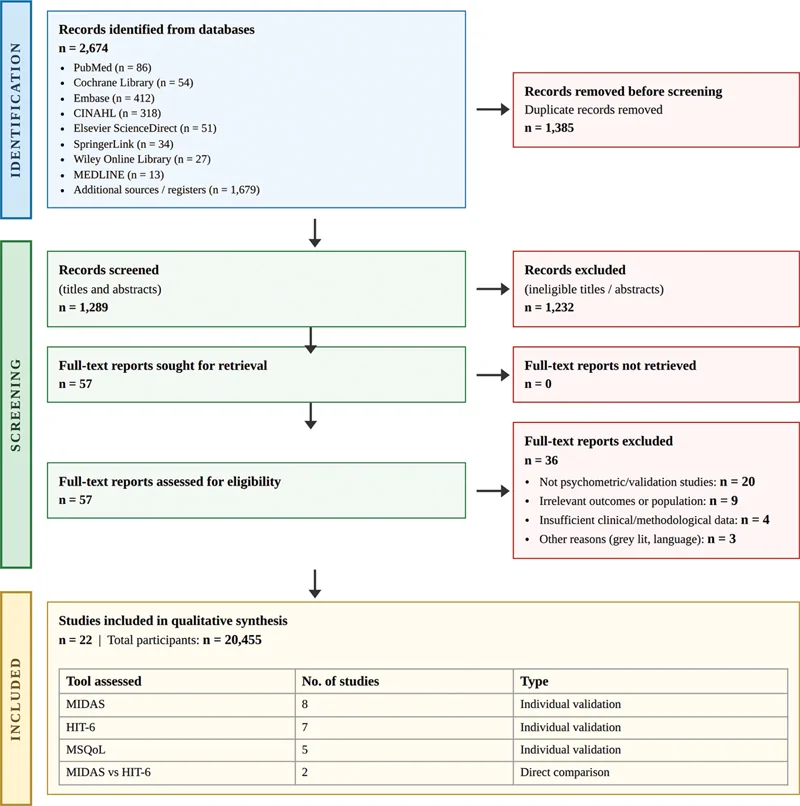
Abbreviations: HIT-6, Headache Impact Test-6; MIDAS, Migraine Disability Assessment Scale; MSQoL, Migraine-Specific Quality-of-Life questionnaire; PRISMA, Preferred Reporting Items for Systematic Reviews and Meta-Analysis. 2020 PRISMAflow diagram illustrating the systematic search and study selection process. Records were identified from eight electronic databases. Following removal of 1,385 duplicates, 1,289 records were screened at the title and abstract stage. Of 57 full-text reports assessed for eligibility, 36 were excluded (nonpsychometric validation studies, n = 20; irrelevant outcomes or population, n = 9; insufficient data, n = 4; other, n = 3). A total of 22 studies were included in the qualitative synthesis (21 identified through database search, 1 Brazilian MIDAS validation added through citation search during peer review). 7

### Study characteristics

Across 22 studies, 20,455 participants were evaluated. From that total, 8 studies assessed MIDAS, 12 13 14 15 16 17 18 19 7 HIT-6, 20 21 22 23 24 25 26 5 MSQoL, 27 28 29 30 31 and 2 compared instruments directly. 32 33 Studies originated from the United States, Iran, Canada, Brazil, Germany, France, Korea, and several European countries. The mean age of participants was 37 to 43 years. The newly added study validated MIDAS in Brazilian Portuguese. 19

### Methodological quality

Under the COSMIN Risk of Bias checklist, most studies were rated very good or adequate for internal consistency, reliability, and structural validity. Some MIDAS and MSQoL studies received doubtful ratings for reliability, owing to small test–retest subsamples and incomplete reporting of measurement error. The methodological quality risk-of-bias analyses are summarized in Figures 2,3 .

**Figure 2:**
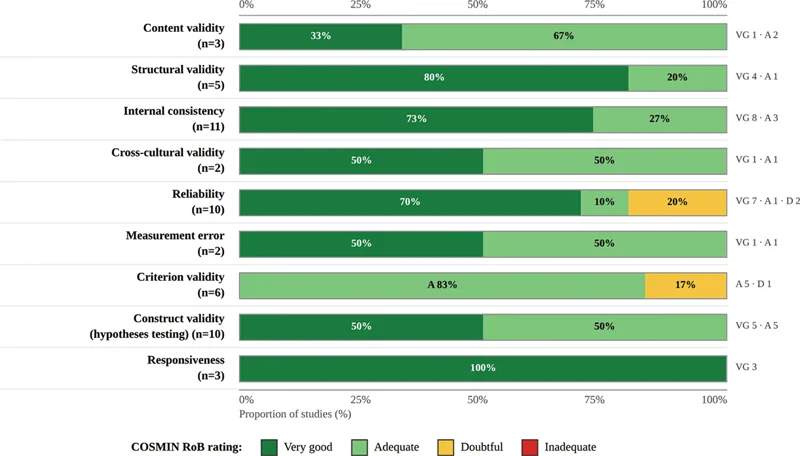
Summary of the methodological quality (risk of bias) of the included studies across the Consensus-Based Standards for the Selection of Health Measurement Instruments (COSMIN) measurement properties, assessed using the COSMIN Risk-of-Bias checklist. Each bar represents the proportion of studies evaluating that property that were rated very good, adequate, doubtful, or inadequate, applying the “worst-score-counts” principle. The number of studies assessing each property (n) is indicated. No property received an “inadequate” rating. Doubtful ratings for reliability arose from small test–retest subsamples (e.g., the Brazilian MIDAS validation) and incomplete reporting. 19

**Figure 3:**
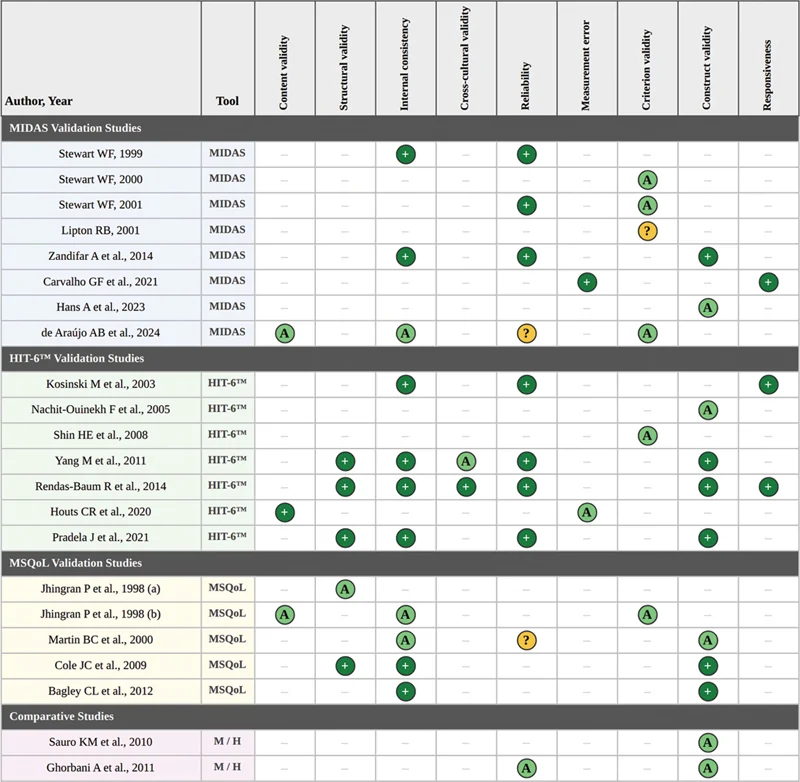
Traffic-light plots of the methodological quality (risk of bias) of each included study, rated per measurement property using the COSMIN Risk-of-Bias checklist. Cells indicate whether a study assessed a giver property and, if so, its methodological quality rating (very good, adequate, doubtful, or inadequate). A dash (-) indicates the property was not assessed in that study. Studies are grouped by the primary instrument evaluated; M/H denotes a MIDAS-HIT-6 comparative study. The Brazilian MIDAS validation received a doubtful reliability rating owing to a small test-retest subsample. 19

### Migraine Disability Assessment (MIDAS)

The use of MIDAS showed sufficient internal consistency in the original and Iranian versions (Cronbach's α = 0.80–0.83), 12 16 as well as sufficient reliability (test–retest r = 0.67–0.80). 12 13 14 Construct validity was sufficient, being convergent with the 36-Item Short-Form Health Survey (SF-36: -0.41 to -0.61). 16 Criterion validity against a diary measure was borderline (r = 0.63, insufficient), 13 and responsiveness was insufficient (AUC = 0.63–0.68). 17 Notably, the Brazilian Portuguese version (n = 149) 19 reported internal consistency (α = 0.655) and intra-rater reliability (ICC = 0.612) below the 0.70 threshold, with satisfactory content validity (content validity index [CVI] = 0.78), revealing inconsistency across cultural adaptations ( Table 1 ). 19

**Table 1:** Characteristics and measurement properties of included MIDAS studies

Author, year	Country	Participants	Mean age (years)	Data collection method	Property tested	Outcome measures	Key results
Stewart et al., 1999 12	United States	97 migraine + 80 non-migraine	38.6	Computer-assisted telephone interview	Test–retest reliability; internal consistency	Missed days from work/household; nonwork activity days	Test–retest r = 0.67–0.73; α = 0.83; MIDAS vs. outcome r = 0.84
Stewart et al., 2000 13	United States	144 migraine patients	NR	90-day diary; self-administered MIDAS	Criterion validity (MIDAS vs. diary)	MIDAS vs diary-based disability	Mean MIDAS 14.53; diary 13.5; r = 0.63
Stewart et al., 2001 14	United States	201 participants	NR	90-day diary; physician assessment	Reliability; criterion validity; clinical significance	Missed days from work/household/nonwork	Test–retest r = 0.80; criterion r = 0.63; clinical r = 0.69
Lipton et al., 2001 15	United States	12 records; 49 physicians	NR	Patient health records	Clinical significance	Pain; disability; need for care	MIDAS vs. physician need-for-care r = 0.69
Zandifar et al., 2014 16	Iran	267 patients	NR	Self-administered, 3- and 8-week follow-up	Internal consistency; reliability; construct validity	MIDAS; SF-36	α = 0.80; test-retest r = 0.54–0.71; convergent r = − 0.41 to − 0.61
Carvalho et al., 2021 17	Germany	103 migraine patients	39.9	Face-to-face; GRoC anchor	Responsiveness; MIC; SEM; SDC	Disability change vs. GRoC	MIC = 4.5; r = 0.29–0.33; AUC = 0.63–0.68 (limited responsiveness)
Hans et al., 2023 18	United States	160 primary headache patients	NR	Face-to-face interview	Construct validity	Resilience vs. MIDAS disability	Negative correlation r = -0.21 ( *p* = 0.009)
Araújo et al., 2024 19	Brazil	149 migraine patients	38	Two-stage cross-cultural validation; test-retest	Content validity; internal consistency; reliability; criterion validity	Brazilian Portuguese MIDAS; HIT-6	CVI = 0.78; α = 0.655; ICC = 0.612 (< 0.70); Weak correlation with HIT-6

Abbreviations: CVI, content validity index; GRoC, global rating of change; HIT-6, Headache Impact Test-6; ICC, intraclass correlation coefficient; MIC, minimal important change; MIDAS, Migraine Disability Assessment; NR, not reported; SDC, smallest detectable change; SEM, standard error of measurement; SF-36, 36-Item Short-Form Health Survey.

### Headache Impact Iest (HIT-6)

The HIT-6 showed sufficient content validity, 25 structural validity, 23 24 26 internal consistency (α = 0.75–0.97), 20 24 26 reliability (ICC = 0.76–0.95), 20 24 26 cross-cultural validity (6-country validation with DIF assessed), 24 and responsiveness (coefficients 0.82–1.00). 20 Correlation with MIDAS was moderate (r = 0.56), as shown in Table 2 .

**Table 2:** Characteristics and measurement properties of included HIT-6 studies

Author, year	Country	Participants	Mean age (years)	Data collection method	Property tested	Outcome measures	Key results
Kosinski et al., 2003 20	United States	1,103 (540 follow-up)	NR	Internet-based survey	Internal consistency; reliability; responsiveness	Headache impact; functioning domains	α = 0.89; test–retest = 0.80; responsiveness: 0.82–1.00; accuracy: 88.7%
Nachit-Ouinekh et al., 2005 21	France	2,537 patients	NR	Face-to-face interview	Construct validity	HIT-6 vs. MIGSEV, QVM	Significant convergent validity reported
Shin et al., 2008 22	Korea	130 migraine without aura	NR	Patient headache diaries	Criterion validity	Diary-based disability vs. HIT-6	Diary 2.7 d/mo; severe HIT-6 4.6 d/mo; moderate 2.6 d/mo
Yang et al., 2011 23	United States	2,049 participants	NR	National headache impact survey	Structural validity; internal consistency; reliability; cross-cultural	Impact across diagnostic groups	α = 0.83–0.87; ICC = 0.77; HIT-6 vs. MIDAS r = 0.56
Rendas-Baum et al., 2014 24	6 countries	1,384 chronic migraine	41	Interventional; baseline + follow-up	CFA; DIF; reliability; responsiveness	Headache impact; ≥ 50% day reduction	α = 0.75–0.92; ICC = 0.76–0.80; convergent -0.86 to -0.59
Houts et al., 2020 25	United States	12 studies; 283 interviews	NA	Qualitative review	Content validity; measurement error	Relevance of HIT-6 items	All six items confirmed relevant
Pradela et al., 2021 26	Brazil	132 (86 primary; 46 secondary)	39.6	Headache OPD appointment	CFA; internal consistency; reliability; construct	HIT-6 Brazil; SF-12	α = 0.97; ICC = 0.95; construct r = -0.64 with SF-12

Abbreviations: CFA, confirmatory factor analysis; DIF, differential item functioning; HIT-6, Headache Impact Test-6; ICC, intraclass correlation coefficient; NA, not applicable; OPD, outpatient department.

### Migraine-Specific Quality-of-Life questionnaire (MSQoL)

The MSQoL showed sufficient structural validity (three-domain CFA), 27 30 internal consistency (α = 0.86–0.96), 29 31 and construct validity (convergent with Patient Health Questionnaire 4 [PHQ-4], MIDAS, HIT-6). 31 However, test–retest reliability was insufficient (ICC = 0.57–0.63), 29 and responsiveness data were limited (indeterminate). Chronic migraine patients reported lower scores than episodic patients ( Table 3 ). 31

**Table 3:** Characteristics and measurement properties of included MSQoL studies

Author, year	Country	Participants	Mean age (years)	Data collection method	Property tested	Outcome measures	Key results
Jhingran et al., 1998 27	United States	351	NR	Self-administered	Structural validity (PCA)	Migraine QoL domains	Factor analysis confirmed construct
Jhingran et al., 1998 28	United States	458	NR	Mailed survey	Internal consistency; content; criterion validity	QoL	Acceptable psychometric properties; valid and reliable
Martin et al., 2000 29	United States	267	NR	Experimental design	Internal consistency; reliability; construct	MSQoL v2.1 vs. SF-36	α = 0.86–0.96; ICC = 0.57–0.63 (< 0.70)
Cole et al., 2009 30	United States	916	NR	Face-to-face interview	CFA; DIF; internal consistency; construct	MSQoL v2.1 vs. SF-36	13-item scale: excellent latent structure; no age/gender bias
Bagley et al., 2012 31	United States	8,726 (499 CM; 8,227 EM)	40.3	Internet-based survey	Internal consistency; construct via PHQ-4, MIDAS, HIT-6	Quality of life (CM vs. EM)	α = 0.90–0.96; lower QoL in CM

Abbreviations: CFA, confirmatory factor analysis; CM, chronic migraine; DIF, differential item functioning; EM, episodic migraine; HIT-6, Headache Impact Test-6; ICC, intraclass correlation coefficient; MIDAS, Migraine Disability Assessment; MSQoL, Migraine-Specific Quality-of-Life questionnaire; NR, not reported; PCA, principal component analysis; PHQ-4, Patient Health Questionnaire-4; QoL, quality of life; SF-36, 36-Item Short-Form Health Survey.

### Comparative analysis

Both HIT-6 and MIDAS were moderately-to-strongly correlated (r = 0.52–0.84), as shown in ( Table 4 ). 32 33 The HIT-6 correlated more strongly with headache intensity (r = 0.46 vs. 0.26) and classified more patients as severely disabled (79 vs. 57%), 32 while MIDAS showed superior test–retest reliability in one cohort. 33 The full COSMIN evidence synthesis, including the quality of evidence and instrument recommendations, is presented in Table 5 .

**Table 4:** Comparative studies of MIDAS and HIT-6

Author, year	Country	Participants	Data collection	Comparison	Outcome measures	Results	Implications
Sauro et al., 2010 32	Canada	798	Headache referral clinic	HIT-6 vs. MIDAS regression	Frequency; intensity; BDI	r = 0.52; HIT-6 stronger with intensity (0.46 vs. 0.26); 79 vs. 57% severe	HIT-6 relates more to intensity; classifies more disability
Ghorbani et al., 2011 33	Iran	240 male migraine patients	Clinic visits, baseline + 3 weeks	HIT-6 vs. MIDAS	Reliability; convergent validity	Both acceptable; strong correlation r = 0.84; MIDAS stronger test–retest	MIDAS superior psychometric performance in this cohort

Abbreviation: BDI, Beck Depression Inventory; COSMIN, Consensus-Based Standards for the Selection of Health Measurement Instruments; GRADE, Grading of Recommendations, Assessments, Development, and Evaluation; HIT-6, headache impact test-6; MIDAS, migraine disability assessment.

**Table 5:** Evidence synthesis for COSMIN: measurement-property ratings, quality of evidence, and recommendations

Measurement property	MIDAS	HIT-6	MSQoL
**Content validity**	+	+	+
**Structural validity**	?	+	+
**Internal consistency**	+, –	+	+
**Cross-cultural validity**	+	+	+
**Reliability**	+, –	+	−
**Measurement error**	?	+	?
**Criterion validity**	−	+	+
**Construct validity (hypotheses)**	+	+	+
**Responsiveness**	−	+	?
**Quality of evidence (GRADE)**	Moderate	Moderate–High	Low–Moderate
**Recommendation**	Category A	Category A	Category B

Abbreviations: COSMIN, Consensus-Based Standards for the Selection of Health Measurement Instruments; GRADE, Grading of Recommendations, Assessments, Development, and Evaluation; HIT-6, headache impact test-6; MIDAS, Migraine Disability Assessment; MSQoL, Migraine-Specific Quality-of-Life questionnaire.

Notes:  + sufficient; − insufficient; ? indeterminate. Category A. recommended for use; B. potential, further research needed; C. not recommended.

## DISCUSSION

This systematic review of 22 studies (20,455 participants) is, to our knowledge, the first to apply the COSMIN framework to a head-to-head appraisal of MIDAS, HIT-6, and MSQoL. By assessing each measurement property, grading the evidence, and formulating recommendations, this review moves beyond a narrative summary to a structured synthesis. Wei et al. 34 and Bae et al. 35 previously evaluated 19 migraine diagnostic questionnaires; our review addresses the complementary domain of impact and disability measurement instruments.

### Migraine Disability Assessment (MIDAS)

Regarding MIDAS, it performed well for internal consistency, reliability, and construct validity in the original, Iranian, and German versions, 12 13 14 15 16 17 but its responsiveness was limited (AUC: 0.63–0.68; minimal important change [MIC]: 4.5), 17 and criterion validity against diary measures was borderline (r = 0.63), likely reflecting 90-day recall bias. Importantly, the Brazilian Portuguese validation reported internal consistency (0.655) and reliability (ICC: 0.612) below threshold, 19 indicating that performance is not uniform across cultural adaptations.

As such, sufficient content validity and internal consistency support a Category A recommendation, with the caveat that MIDAS is better suited to cross-sectional disability quantification than to tracking treatment response.

### Headache Impact Test (HIT-6)

The HIT-6 achieved sufficient ratings across every property, with the strongest evidence base. Kosinski et al. established internal consistency and responsiveness. 20 Yang et al. and Rendas-Baum et al. confirmed structural validity and cross-cultural invariance. 23 24 Houts et al. confirmed content validity. 25 Finally, Pradela et al. reported excellent reliability in Brazil (α = 0.97; ICC: 0.95). 26 Its brevity and breadth—validated across primary and secondary headache disorders—make HIT-6 ideal for rapid screening and warrant a Category A recommendation.

### Migraine-Specific Quality-of-Life questionnaire (MSQoL)

Finally, MSQoL uniquely captures migraine-specific quality of life. Its structural validity, internal consistency, and construct validity were sufficient, 27 29 30 31 and Cole et al. demonstrated an optimal 13-item structure free of age or gender bias. 30 However, test–retest reliability was insufficient (ICC: 0.57–0.63) 29 and responsiveness evidence was limited, yielding a Category B recommendation pending further reliability and responsiveness research.

### Complementarity and clinical implications

The instruments measure distinct constructs and are complementary. The HIT-6 suits brief, broad screening; MIDAS quantifies disability and lost productivity for occupational and insurance assessments; MSQoL adds psychosocial and QoL information for patient-centered care. Moderate intertool correlations (r = 0.52–0.84) 32 33 confirm shared variance without redundancy. Seo et al. demonstrated convergence with mental-health measures, 36 37 underscoring the value of multi-instrument assessment.

Cross-cultural validation across Korean, French, Turkish, Hindi, Arabic, Spanish, and Brazilian Portuguese populations 19 38 39 40 41 42 43 generally supported transferability of the underlying constructs, though some adaptations (e.g., Brazilian Portuguese MIDAS) showed reduced reliability, highlighting the need for population-specific psychometric verification.

### Strengths and limitations

The principal strength is the COSMIN-based methodology enabling transparent, property-by-property appraisal and graded recommendations. Limitations include the English-language restriction (language bias), heterogeneity in reference standards and recall periods, and incomplete reporting of measurement error in some studies. Future research should prioritize head-to-head studies with identical reference standards, formal responsiveness and measurement-error analyses (especially for MIDAS and MSQoL), and digital administration.

In conclusion, both HIT-6 and MIDAS can be recommended for assessing migraine impact (Category A), with HIT-6 offering the most robust evidence and MIDAS excelling at disability quantification despite limited responsiveness. As for MSQoL, it provides valuable QoL information but requires further reliability and responsiveness research (Category B). A combined, context-driven approach is likely to yield the most comprehensive evaluation of migraine burden in clinical and research settings.
